# Supplementary material for: A Novel Prognostic DNA Methylation Panel for Colorectal Cancer
Source: Int J Mol Sci. 2019 Sep 20;20(19):4672. doi: 10.3390/ijms20194672 (PMC6801964; doi:10.3390/ijms20194672)
Supplement: Supplementary file 1 [file ijms-20-04672-s001.pdf]

International Journal of
Molecular Sciences

MDPI

Supplementary data

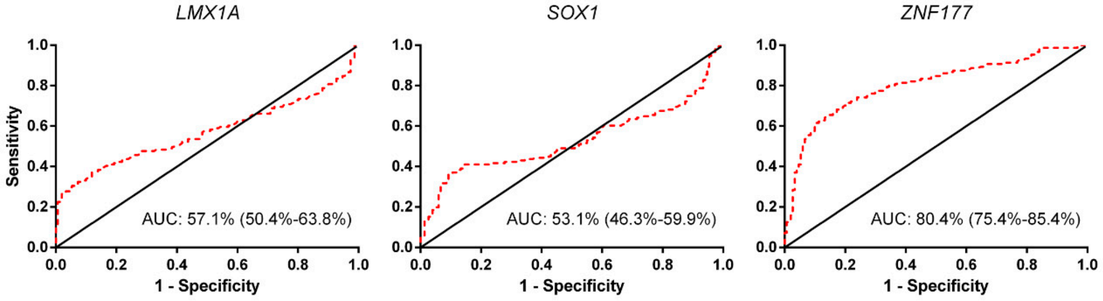

Figure S1. ROC curves of *LMX1A*, *SOX1* and *ZNF177* in CRC tissues. ROC curves were generated to determine the optimal cut-off point of *LMX1A*, *SOX1* and *ZNF177* methylation in discriminating 151 paired CRC tissues and the nontumor tissues.

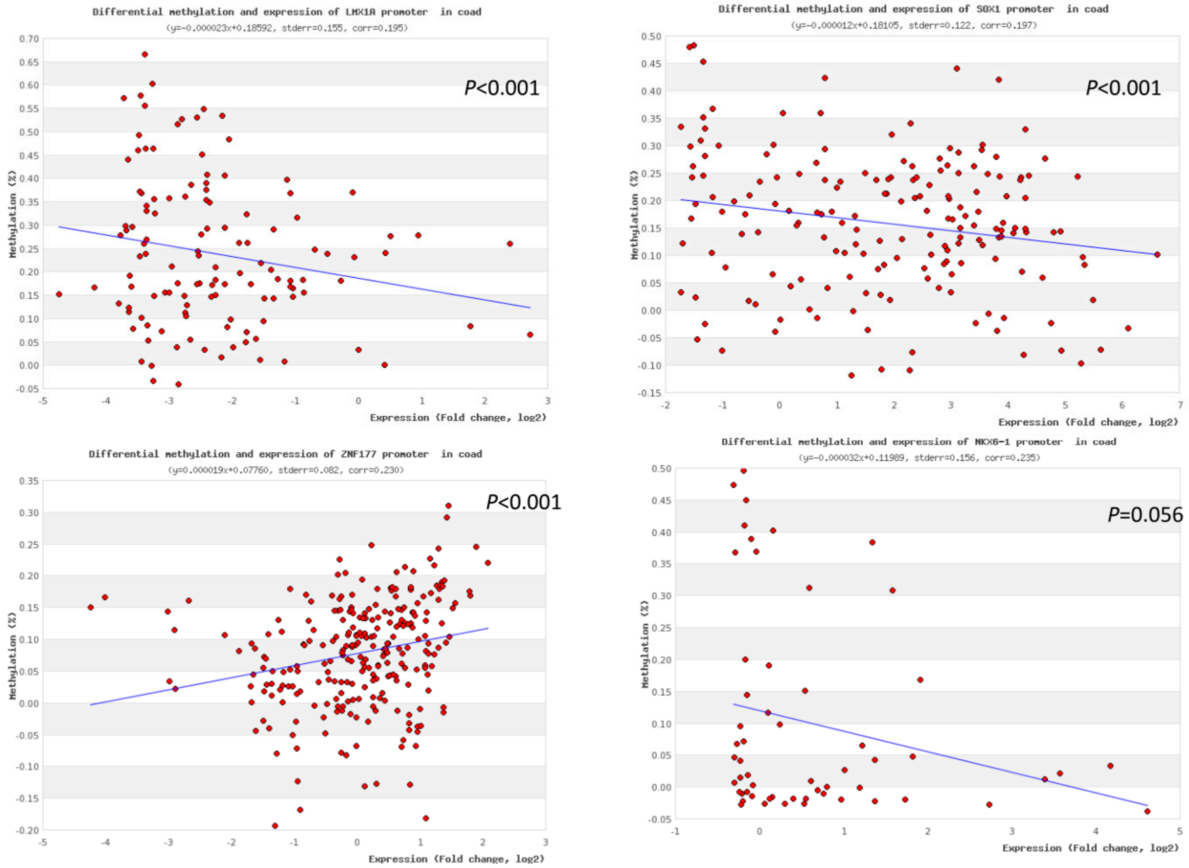

Figure S2. Correlation between gene expression levels and methylation status of *LMX1A*, *SOX1*, *ZNF177* and *NKX6.1* in CRC tissues. The differential methylation and expression levels of *LMX1A*, *SOX1*, *ZNF177* and *NKX6.1* in colon adenocarcinoma patients was based on data derived from the MethHC database (http://MethHC.mbc.nctu.edu.tw). The inverse correlation between the gene expression and DNA methylation of *SOX1* (correlation=-0.2971, p<0.0001) was statistically significant. There was no correlation between the methylation and gene expression of *LMX1A* (correlation=-0.00675, p<0.0001) and *NKX6.1* (correlation=-0.1046, p=0.0566) from the MethHC database. However, a positive correlation was observed between the gene expression level and the methylation status of for *ZNF177* (correlation=0.3487, p<0.0001).

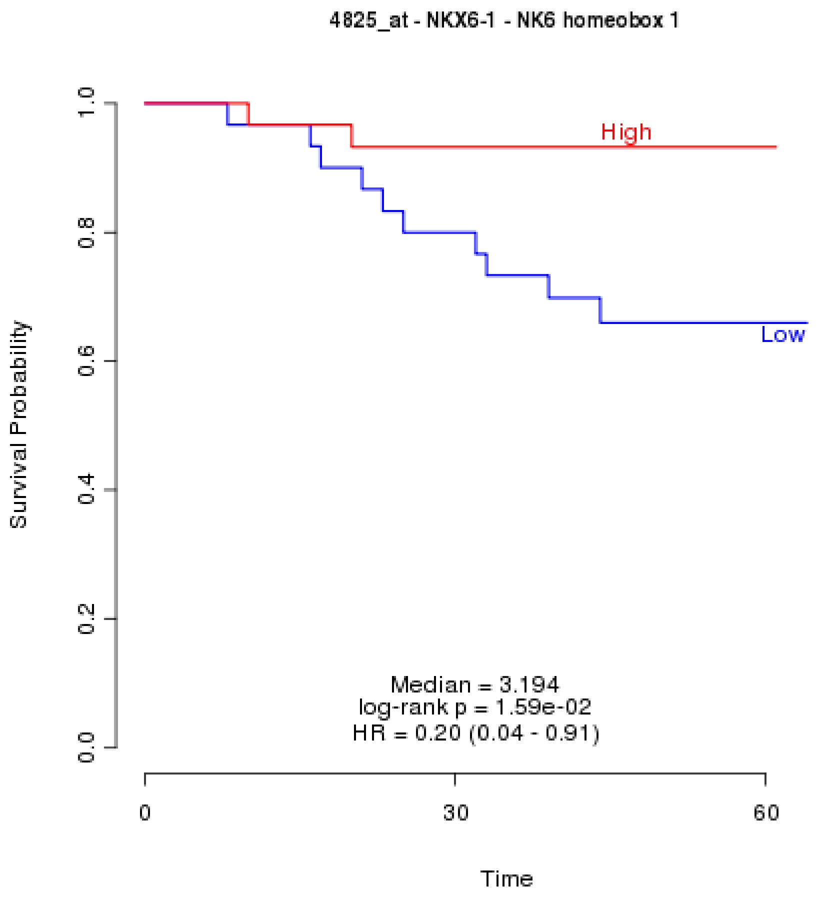

Figure S3. Kaplan-Meier curves for survival analysis in 62 CRC patients**.** The overall survival and disease-free survival rates of CRC patients with different *NKX6.1* expression statuses are presented. Red lines indicate cases with high *NKX6.1* expression. Blue lines indicate cases without *NKX6.1* expression. The dataset (GSE12945) showed that patients with low *NKX6.1* expression had poorer survival compared with those with high *NKX6.1* expression (p=0.0159).

Table S1. Univariate and multivariate of overall survival analysis using clinical characteristics and the *LMX1A* methylation panel in 151 CRC patients

Variable	Univariate analysis Hazard ratio (95% confidence interval)	Multivariate analysis Hazard ratio (95% confidence interval)
Age (years)	0.99 (0.96-1.02)	0.98 (0.95-1.01)
Sex (female vs male)	1.05 (0.49-2.22)	1.28 (0.54-3.04)
LMX1A methylation		
Unmethylation	Reference	Reference
Methylation	**2.58 (1.18-5.65)***	**3.30 (1.25-8.76)***
Stage		
I+II	Reference	Reference
III+IV	1.77 (0.81-3.87)	2.71 (0.99-7.40)
Tumor grade		
Well + moderately	Reference	Reference
Poorly or undifferentiated	0.67 (0.16-2.86)	0.59 (0.13-2.73)
Tumor size		
≦5 cm	Reference	Reference
＞5 cm	0.97 (0.42-2.21)	0.71 (0.28-1.77)
No. of lymph node		
≧12	Reference	Reference
0-11	0.42 (0.17-1.07)	0.49 (0.17-1.47)
Chemotherapy		
No	Reference	Reference
Yes	0.51 (0.23-1.13)	**0.33 (0.12-0.87)***

*P <0.05, **P <0.01.

Table S2. Univariate and multivariate of overall survival analysis using clinical characteristics and the *SOX1* methylation panel in 151 CRC patients

Variable	Univariate analysis Hazard ratio (95% confidence interval)	Multivariate analysis Hazard ratio (95% confidence interval)
Age (years)	0.99 (0.96-1.02)	0.99 (0.96-1.02)
Sex (female vs male)	1.05 (0.49-2.22)	1.27 (0.54-2.99)
SOX1 methylation		
Unmethylation	Reference	Reference
Methylation	1.90 (0.72-5.02)	2.26 (0.80-6.42)
Stage		
I+II	Reference	Reference
III+IV	1.77 (0.81-3.87)	**2.77 (1.05-7.29)***
Tumor grade		
Well + moderately	Reference	Reference
Poorly or undifferentiated	0.67 (0.16-2.86)	0.68 (0.15-3.06)
Tumor size		
≦5 cm	Reference	Reference
＞5 cm	0.97 (0.42-2.21)	0.81 (0.32-2.00)
No. of lymph node		
≧12	Reference	Reference
0-11	0.42 (0.17-1.07)	0.45 (0.16-1.31)
Chemotherapy		
No	Reference	Reference
Yes	0.51 (0.23-1.13)	**0.37 (0.15-0.96)***

*P <0.05, **P <0.01.

Table S3. Univariate and multivariate of overall survival analysis using clinical characteristics and the *ZNF177* methylation panel in 151 CRC patients

Variable	Univariate analysis Hazard ratio (95% confidence interval)	Multivariate analysis Hazard ratio (95% confidence interval)
Age (years)	0.99 (0.96-1.02)	0.99 (0.96-1.02)
Sex (female vs male)	1.05 (0.49-2.22)	1.13 (0.48-2.65)
ZNF177 methylation		
Unmethylation	Reference	Reference
Methylation	1.72 (0.59-5.00)	1.58 (0.44-5.75)
Stage		
I+II	Reference	Reference
III+IV	1.77 (0.81-3.87)	**3.00 (1.13-7.98)***
Tumor grade		
Well + moderately	Reference	Reference
Poorly or undifferentiated	0.67 (0.16-2.86)	0.69 (0.15-3.21)
Tumor size		
≦5 cm	Reference	Reference
＞5 cm	0.97 (0.42-2.21)	0.85 (0.34-2.12)
No. of lymph node		
≧12	Reference	Reference
0-11	0.42 (0.17-1.07)	0.48 (0.16-1.40)
Chemotherapy		
No	Reference	Reference
Yes	0.51 (0.23-1.13)	**0.33 (0.13-0.86)***

*P <0.05, **P <0.01.

Table S4. Univariate and multivariate of overall survival analysis using clinical characteristics and the *NKX6.1* methylation panel in 151 CRC patients

Variable	Univariate analysis Hazard ratio (95% confidence interval)	Multivariate analysis Hazard ratio (95% confidence interval)
Age (years)	0.99 (0.96-1.02)	0.97 (0.94-1.00)
Sex (female vs male)	1.05 (0.49-2.22)	1.44 (0.61-3.43)
NKX6.1 methylation		
Unmethylation	Reference	Reference
Methylation	**2.60 (1.18-5.72)****	**6.06 (2.18-16.88)****
Stage		
I+II	Reference	Reference
III+IV	1.77 (0.81-3.87)	**3.77 (1.25-11.36) ***
Tumor grade		
Well + moderately	Reference	Reference
Poorly or undifferentiated	0.67 (0.16-2.86)	0.47 (0.10-2.25)
Tumor size		
≦5 cm	Reference	Reference
＞5 cm	0.97 (0.42-2.21)	0.60 (0.23-1.57)
No. of lymph node		
≧12	Reference	Reference
0-11	0.42 (0.17-1.07)	0.43 (0.15-1.25)
Chemotherapy		
No	Reference	Reference
Yes	0.51 (0.23-1.13)	**0.26 (0.09-0.73)***

*P <0.05, **P <0.01.

Table S5. Primer Sequences used for RT-PCR

LMX1A-F	TCAGAAGGGTGATGAGTTTGTCC
LMX1A-R	GGGGCGCTTATGGTCCTTG
ZNF177-F	GGAATTTTACAAGGTGACTGTGC
ZNF177-R	AGAGTGAGTTCTGACACAGTTCGT
SOX1-F	GAGATTCATCTCAGGATTGAGATTCTA
SOX1-R	GGCCTACTGTAATCTTTTCTCCACT
